# Dynasore blocks evoked release while augmenting spontaneous synaptic transmission from primary visceral afferents

**DOI:** 10.1371/journal.pone.0174915

**Published:** 2017-03-30

**Authors:** Mackenzie E. Hofmann, Michael C. Andresen

**Affiliations:** Department of Physiology & Pharmacology, Oregon Health & Science University, Portland, Oregon, United States of America; The Research Center of Neurobiology-Neurophysiology of Marseille, FRANCE

## Abstract

The recycling of vesicle membrane fused during exocytosis is essential to maintaining neurotransmission. The GTPase dynamin is involved in pinching off membrane to complete endocytosis and can be inhibited by dynasore resulting in activity-dependent depletion of release-competent synaptic vesicles. In rat brainstem slices, we examined the effects of dynasore on three different modes of glutamate release–spontaneous, evoked, and asynchronous release–at solitary tract (ST) inputs to neurons in the nucleus of the solitary tract (NTS). Intermittent bursts of stimuli to the ST interspersed with pauses in stimulation allowed examination of these three modes in each neuron continuously. Application of 100 μM dynasore rapidly increased the spontaneous EPSC (sEPSC) frequency which was followed by inhibition of both ST-evoked EPSCs (ST-EPSC) as well as asynchronous EPSCs. The onset of ST-EPSC failures was not accompanied by amplitude reduction–a pattern more consistent with conduction block than reduced probability of vesicle release. Neither result suggested that dynasore interrupted endocytosis. The dynasore response profile resembled intense presynaptic TRPV1 activation. The TRPV1 antagonist capsazepine failed to prevent dynasore increases in sEPSC frequency but did prevent the block of the ST-EPSC. In contrast, the TRPV1 antagonist JNJ 17203212 prevented both actions of dynasore in neurons with TRPV1-expressing ST inputs. In a neuron lacking TRPV1-expressing ST inputs, however, dynasore promptly increased sEPSC rate followed by block of ST-evoked EPSCs. Together our results suggest that dynasore actions on ST-NTS transmission are TRPV1-independent and changes in glutamatergic transmission are not consistent with changes in vesicle recycling and endocytosis.

## Introduction

To sustain synaptic transmission, exocytotic vesicle release must be balanced with restoration of the pool of ready-releasable vesicles. Regenerating vesicles requires an endocytotic step in which membrane is retrieved and recycled to generate new vesicles in a timely fashion. Key aspects of these processes are calcium dependent and different forms of transmission likely engage multiple pools of vesicles [1–4]. The small molecule, dynasore, selectively and reversibly interrupts membrane endocytosis by inhibition of dynamin and thus vesicle recycling [5, 6]. Block of endocytosis by dynasore leads to vesicle depletion and produces vesicle component accumulation at the surface membrane in an activity dependent manner [7]. Dynasore reduces evoked response amplitudes independent from spontaneous release suggesting differential actions across release modes [8]. Thus, dynasore discriminated between activity-dependent and activity-independent synaptic vesicle release.

In cranial visceral afferent reflexes, peripheral primary sensory neurons send central processes to form synaptic terminals within the nucleus of the solitary tract (NTS) [9–11]. Most cranial primary afferent neurons have unmyelinated peripheral axons that form the solitary tract (ST) and express transient receptor potential vanilloid type 1 receptors (TRPV1) on their central synaptic terminals [9, 12, 13]. TRPV1 serves as a unique source of calcium influx which drives afferent basal glutamate vesicle release independent of voltage activated calcium channels (VACCs) onto NTS second order neurons [4]. Thus, ST synapses formed by unmyelinated axons feature both VACC-dependent and VACC-independent vesicle release [4, 14, 15]. Activation of TRPV1 with moderate temperatures or vanilloid agonist triggered increased spontaneous release of glutamate (sEPSCs) without altering ST-evoked excitatory postsynaptic current (ST-EPSC) amplitudes [14, 16]. A third mode of vesicle release, asynchronous release, is evident as a transient increase in the frequency of sEPSCs trailing the ST-evoked EPSC [17]. Evoked, spontaneous and asynchronous release of glutamate appear to rely on separate presynaptic domains with unique release characteristics [4]. Here, we tested whether dynasore might separately manipulate activity-dependent, ST-evoked release differently than spontaneous release and yield a better understanding of TRPV1 mediated release.

To test this, we measured evoked, spontaneous and asynchronous release at NTS neurons and followed the time course of dynasore induced changes in synaptic responses. Surprisingly, we found no evidence of the expected, activity-dependent depletion of vesicles. Instead, dynasore paradoxically and rapidly accelerated the rate of spontaneous release while ST-evoked release was blocked entirely. Blockade of evoked ST transmission showed the signs consistent with conduction block rather than amplitude depression. Thus, our studies identify dynasore actions via non-endocytotic mechanisms in ST-NTS transmission.

## Materials and methods

All animal procedures were approved by the Institutional Animal Care and Use Committee at Oregon Health and Science University and conformed to animal welfare guidelines issued by the National Institutes of Health publication *Guide for the Care and Use of Laboratory Animals*.

### Slice preparation

Brainstem slices were obtained from adult (>130 g) male Sprague-Dawley rats (Charles River Laboratories, Wilmington, MA) as previously described in detail [18]. After deep anesthesia (3% isoflurane), the brainstem was removed and placed into ice-cold artificial cerebrospinal fluid (ACSF, see below). Tilting the brainstem allowed for the cutting of a horizontal brainstem slice containing 1–3 mm of the ST in the same plane as the NTS. The brainstem was mounted on a vibrating microtome (VT1000 S; Leica Microsystems, Bannockburn, IL) and slices cut using a sapphire blade (Delaware Diamond Knives, Wilmington, DE). Immediately after obtaining a slice, it was submerged in a recording chamber containing ACSF consisting of, in mM: 125 NaCl, 3 KCl, 1.2 KH_2_PO_4_, 1.2 MgSO_4_, 25 NaHCO_3_, 10 glucose, and 2 CaCl_2_, bubbled with 95% O_2_-5% CO_2_. While being continuously perfused (1.6–2.0 mL/min), an in-line heating system (TC2BIP with HPRE2HF and TH-10Km bath probe; Cell MicroControls, Norfolk, VA) controlled the bath temperature set to 32°C and was continuously monitored downstream of the slice.

### Patch-clamp recording

Whole-cell patch clamp recordings were performed on neurons in the medial NTS within 250 μm rostral of obex and medial to the ST. Neurons were visualized with an infrared differential interference contrast microscope (Zeiss Axioskop FS2). Recordings were made with patch electrodes (2–4 MΩ) pulled from borosilicate glass and filled with an intracellular solution composed of, in mM: 6 NaCl, 4 NaOH, 130 K-Gluconate, 11 EGTA, 2 CaCl_2_, 2 MgCl_2_, 10 HEPES, 2 Na_2_-ATP, 0.2 Na_2_-GTP, pH adjusted to 7.3–7.32. Neurons were voltage clamped at -60 mV with a Multiclamp 700B and pClamp 9.2 software (Molecular Devices, Sunnyvale, CA). Synaptic currents were sampled at 20 kHz and digitally filtered at 10 kHz. Liquid junction potentials were corrected. All recordings were performed in the presence of gabazine (SR-95531, 3 μM in ACSF) and, at the end of the experiments, the TRPV1 agonist resiniferatoxin (RTX, 1 nM) was applied. Dynasore (100 μM), RTX, and two TRPV1 antagonists, capsazepine (CPZ, 10 μM), and JNJ 17203212 (JNJ, 10 μM) were dissolved in 100% ethanol but on dilution, the final concentration of ethanol in ACSF did not exceed 2 μl/ml. Ethanol vehicle at final concentration had no effect on synaptic responses. A subset of experiments included tetrodotoxin (TTX, 1 μM) to block voltage gated sodium channels. All drugs were purchased from R&D Systems.

### Afferent activation and identification

Synaptic responses were evoked via a concentric bipolar stimulating electrode (200 μm outer tip diameter; Frederick Haer Co., Bowdoinham, ME) placed on the ST at least 1 mm rostral from the recorded medial NTS neurons. Bursts of five shocks (100 μs duration at 50 Hz) elicited evoked ST-EPSCs every 6 sec or 10 sec. This standard protocol allowed for the collection of ST-EPSCs together with pre-shock samples to assess basal sEPSCs as well post stimulation samples to gauge asynchronous release [19]. The latency from the stimulus shock to the onset of the first EPSC evoked in each burst was measured and averaged across ≥ 30 ST shocks. The jitter was calculated as the standard deviation of the latency [18] and jitter values < 200 μs indicated that these are monosynaptic inputs [20]. Neurons which received only high jitter responses (>200 μs) were considered higher-order and not studied further. The presence of asynchronous EPSCs following bursts of ST shocks and/or a response to a thermal challenge was indicative of TRPV1+ classification [17, 18, 21, 22].

### Data analysis and statistics

Analysis was performed using O-phys [23] and Origin 2016 (OriginLab; Northampton, MA). The spontaneous activity was measured over either the 1 sec or 5 sec prior to the stimulation depending on the inter-sweep interval (6 sec or 10 sec sweeps, respectively). From these same recordings, the amplitudes and failure rates of the first ST-EPSC were assessed. Failures were detected by examining the interval of time following ST shocks in which the EPSC was expected to arrive and plotted as zero values in amplitude diary plots if failures occurred. Failure rate was calculated as the number of successes divided by the number of test shocks. Responses of EPSCs 2–5 were not assessed to avoid use dependent failures [24]. Both evoked (ST-EPSCs) and spontaneous release (sEPSCs) were analyzed over 3 minutes in each condition (e.g., control, dynasore, and wash). For statistical comparisons, the Kolmogorov-Smirnov (KS) test examined interevent intervals and amplitudes of sEPSCs within cells. Group data were analyzed with either paired t-tests or repeated measures (RM) ANOVA with Tukey post hoc comparisons where appropriate. All statistics reported from RM-ANOVAs represent the p-values from the post hoc analyses.

## Results

### Three modes of ST vesicle release

**Fig 1:**
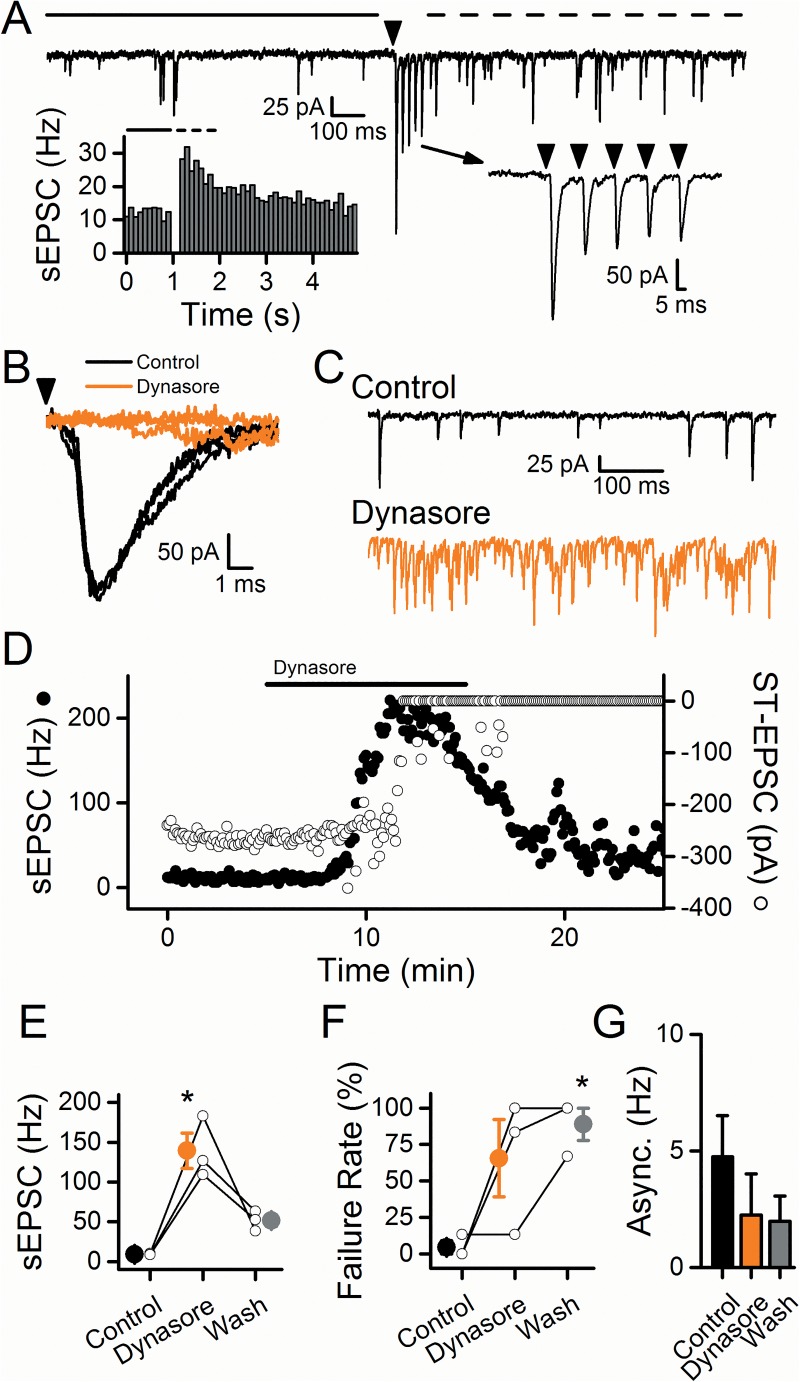
Dynasore increased spontaneous release followed by an inhibition of evoked release at TRPV1+ afferents. In A-D, the data come from a single representative cell. A) Bursts of five stimuli at 50 Hz (arrowhead) resulted in a transient increase in the spontaneous activity over the 1 s after stimulation (dashed line) compared to the 1 s before stimulation (solid line). The left inset portrays this transient increase in sEPSC frequency, termed asynchronous release, which is indicative of the presence of TRPV1. The right inset shows the frequency dependent depression of the evoked ST-EPSCs with each arrowhead representing stimulation of the ST. B) Application of 100 μM dynasore (orange) caused the complete block of the ST-EPSC compared to control (black). C) Dynasore (orange) increased the sEPSC frequency compared to control (black). D) Diary plot depicting the increased sEPSC frequency (closed circles) and inhibition of the ST-EPSC (open circles). Note the spontaneous activity increased well before the ST-EPSC became inhibited and that the evoked response did not recover following washout. Over 3 cells, dynasore significantly increased the sEPSC frequency (E), significantly increased the failure rate of the ST-EPSCs (F), and decreased the asynchronous release (G). Asterisks represent p < 0.05.

Suprathreshold shocks to the ST evoked ST-EPSCs with a consistent latency (jitters <200 μs within neurons) indicative of a monosynaptic connection to second-order NTS neurons, and ST-EPSC responses maintained stable amplitudes over extended periods of this intermittent 50 Hz ST activation [18]. A transient increase in the sEPSC frequency following ST-stimulation, i.e. asynchronous release, trailed the ST-EPSC evoked responses (Fig 1A). This asynchronous release provided initial indication of ST afferent type as TRPV1+ [17]. Neurons with asynchronous release were selected for this study to compare the effects of dynasore on spontaneous, evoked, and asynchronous modes of vesicle release.

### Dynasore differentially impacts evoked and spontaneous release

Dynasore is a cell-permeable inhibitor of dynamin GTPase activity and thus interrupts dynamin-dependent endocytosis [25]. In NTS recordings, the first sign of dynasore (100 μM) action was most often a dramatic increase in sEPSC frequency consistent with an increased probability of release from the spontaneous vesicle pool (Fig 1C–1E). Often lagging slightly behind, the ST-evoked EPSCs began to falter before failing completely with no evoked recovery on prolonged wash (Fig 1D, unfilled circles). Surprisingly, this failure of ST-EPSCs occurred without substantial amplitude degradation preceding full failures–a pattern that is more likely indicative of failed axonal transmission rather than actions on the release mechanism [15]. In our NTS neurons, the dramatic increases in the frequency of spontaneous release persisted for minutes (Fig 1D) and reversed more slowly in prolonged washing in Control solution. However, the ST-EPSCs were not restored by wash suggesting differential actions on spontaneous compared to evoked release (Fig 1D). On average (n = 3), dynasore increased the sEPSC rate by 1518% (Fig 1E, p = 0.008, RM-ANOVA) which returned to control rates on washing (Fig 1F, p = 0.23, RM-ANOVA). In contrast, the increase in failure rates persisted even in wash (Fig 1F; n = 3; p = 0.04; RM-ANOVA) and asynchronous release remained at low levels throughout the wash period (Fig 1G, n = 3, p = 0.49, RM-ANOVA). Thus, the actions of dynasore observed in NTS neurons were quite different than might be expected if blockade of vesicle endocytosis were responsible. Rather, dynasore actions on these ST-NTS neurons resembled the synaptic response profile of intense TRPV1 activation [16, 17].

### Dynasore acts presynaptically on ST terminals

**Fig 2:**
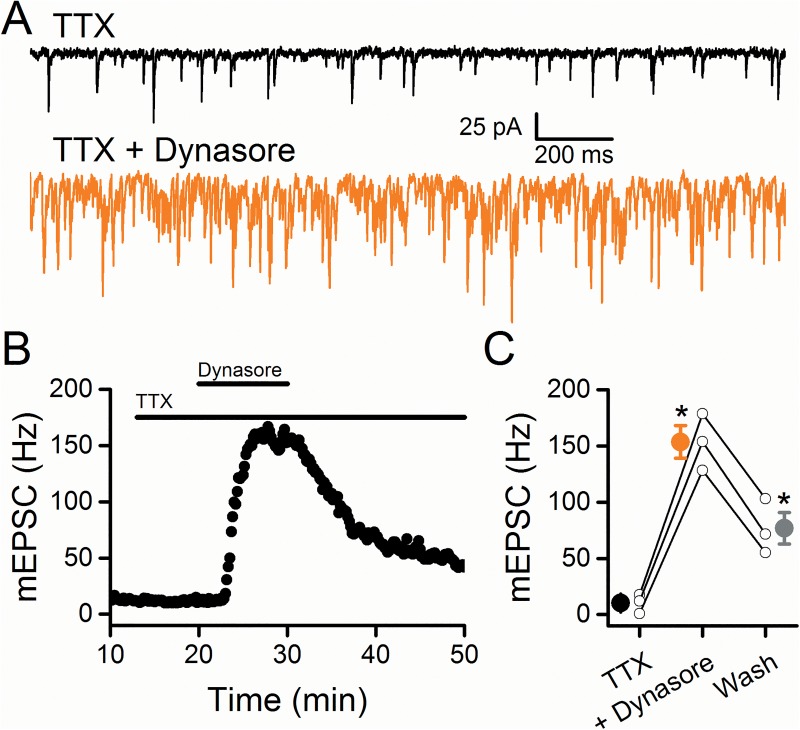
Miniature EPSCs increased following the application of dynasore. Tetrodotoxin (TTX) blocked sodium channels in order to investigate the effect of dynasore on mEPSCs. A) In a representative cell, 100 μM dynasore significantly increased mEPSC frequency (orange) compared to control (black). B) Diary plot of the cell in A displays the massive increase in mEPSC frequency that remained elevated during washout of dynasore. C) On average (n = 3), dynasore significantly increased the frequency of mEPSCs that did not return to control levels upon washout. Asterisks represent p < 0.05.

The classic test of presynaptic sites of action examines miniature synaptic events (mEPSCs) in which conduction is blocked using TTX. In the presence of TTX, dynasore rapidly increased mEPSC rates in NTS neurons (Fig 2, n = 3, p < 0.001; RM-ANOVA) which remained elevated after washing in Control solutions (n = 3, p = 0.003, RM-ANOVA). Thus, even with the voltage gated aspects of synaptic transmission blocked by TTX, the pattern of changes in mEPSCs closely resembled those without TTX suggesting that dynasore acts at presynaptic sites to increase activation of the vesicle release machinery without action potentials.

**Fig 3:**
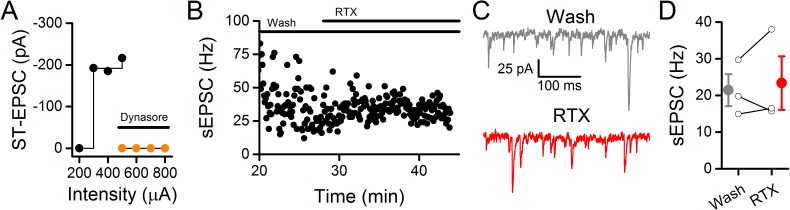
The TRPV1 agonist, resiniferatoxin (RTX), failed to increase the frequency of sEPSCs following application and washout of dynasore. The representative cell in A-C is the same cell from Fig 1. A) With gradual increases to stimulation intensity under Control conditions, the ST-EPSC went from unresponsive to a monosynaptic input (control, black). The amplitude of the ST-EPSC did not change with increased stimulation intensity consistent with a monosynaptic input. In contrast, following dynasore application (orange) increasing the stimulation intensity was unable to obtain an evoked ST-EPSC. B) Following the washout of dynasore, application of 10 nM RTX did not increase the sEPSC frequency. C) Raw data displaying no change to sEPSC activity with RTX (red) compared to the dynasore wash period (gray). D) On average (n = 3), RTX did not increase the frequency of sEPSCs following washout of dynasore.

An additional potential presynaptic site of action was suggested by the observed dynasore block of evoked EPSCs. Such failures in ST conduction resembled the pattern of delayed EPSC arrival induced by the local anesthetic QX-314 [15]. In those experiments, higher intensity ST shocks “rescued” failing ST-EPSCs and we attempted the same strategy during dynasore exposure (Fig 3A). During washout of dynasore using Control solution, increasing ST shock intensity failed to restore the ST-EPSC even upon more than doubling the ST shock intensity (Fig 3A). This result suggests that, unlike QX-314, dynasore is unlikely to simply shift the spike gating threshold within ST axons to induce ST-EPSC failures. The differences between dynasore actions on NTS spontaneous/miniature release and evoked ST-EPSCs are consistent with separate, distinct actions on each process.

### Dynasore prevents vanilloid activation of TRPV1

TRPV1 is strongly linked to spontaneous sEPSC rates in NTS neurons, and vanilloid agonists strongly increase the rate of sEPSCs [12, 14, 17, 26]. In NTS neurons, dynasore augmented sEPSC rates while blocking ST-EPSCs (Fig 1), a pattern identical to strong TRPV1 activation and suggested that promotion of TRPV1 activation might be responsible [4, 16]. Surprisingly however, exposure to dynasore followed by prolonged washout prevented high concentrations (1 nM) of the ultrapotent TRPV1 agonist resiniferatoxin (RTX) from increasing sEPSC rates (Fig 3B–3D, n = 3, p = 0.64, paired t-test). We hypothesized two possible mechanisms for dynasore prevention of RTX activation of TRPV1: dynasore acts directly on TRPV1 to disable/desensitize the channel or indirectly occludes vanilloid activation of TRPV1 by generating a potent endovanilloid. Note that either mechanism represents an action of dynasore independent of endocytotic block.

### Does dynasore alter TRPV1 function?

**Fig 4:**
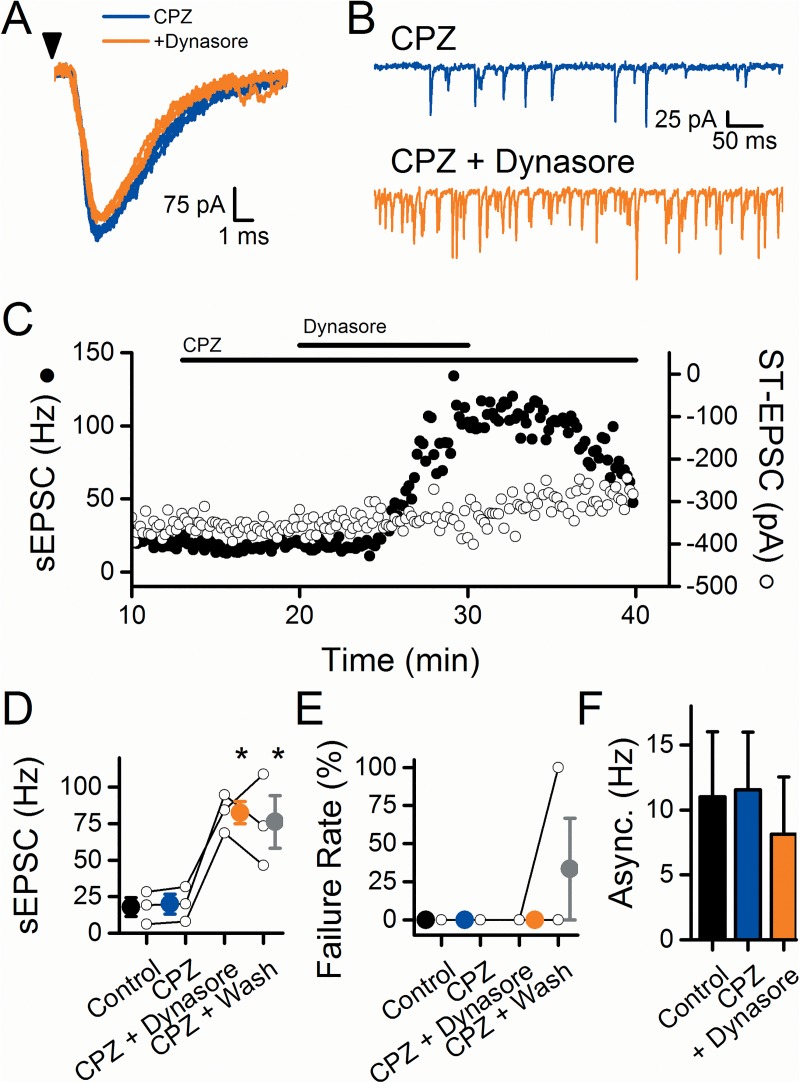
The TRPV1 antagonist capsazepine (CPZ) prevents the dynasore-induced inhibition of the evoked response but not the increase to the spontaneous activity. Panels A-C are from a single representative cell. A) In the presence of CPZ, dynasore (orange) failed to inhibit the evoked response (CPZ, blue). Arrowhead represents stimulation of the ST. B) Dynasore increased the sEPSC rate in the presence of CPZ (CPZ, blue; dynasore, orange). C) Diary plot portrays the increase to sEPSC frequency (closed circles), while the evoked ST-EPSC remained unaltered (open circles). In the presence of CPZ, on average (n = 3) dynasore significantly increased the frequency of sEPSCs (D) but prevented both the increased failure rate of the ST-EPSC (E) and decrease in asynchronous release (F). Asterisks represent p < 0.05.

**Fig 5:**
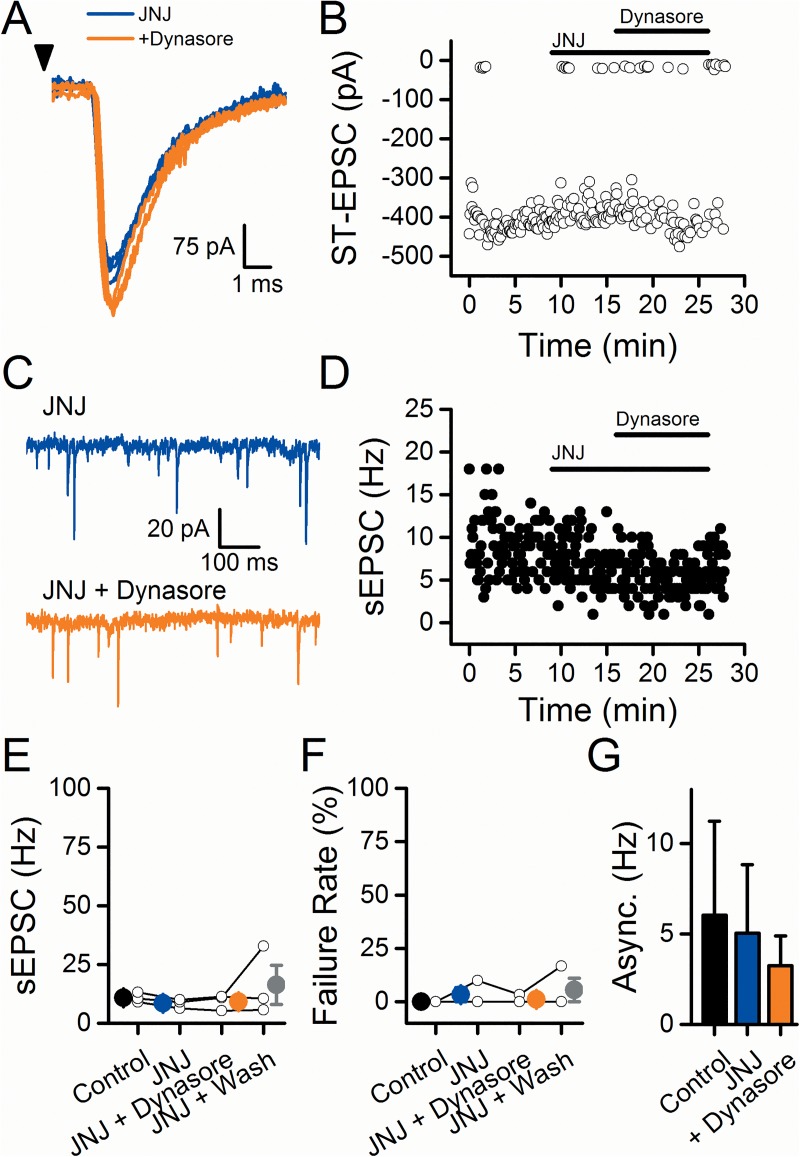
The TRPV1 antagonist JNJ 17203212 (JNJ) prevented the dynasore-induced increase to sEPSC frequency and the inhibition of the ST-EPSC. A single representative cell is depicted in A-D. A) Dynasore did not inhibit the ST-EPSC in the presence of JNJ (JNJ, blue; dynasore, orange). Arrowhead represents stimulation of the ST. B) Diary plot showing that dynasore does not inhibit the ST-EPSC when applied in the presence of JNJ. C) The spontaneous activity did not increase with dynasore in the presence of JNJ (JNJ, blue; dynasore, orange). D) Diary plot demonstrating that JNJ prevented the dynasore-induced increase to the sEPSC frequency. On average (n = 3), JNJ prevented dynasore from increasing the sEPSC frequency (E), increasing the ST-EPSC failure rate (F), and decreasing the asynchronous release (G).

If dynasore generates an endovanilloid, then a competitive TRPV1 antagonist should block the effects. To test this, we treated neurons with the TRPV1 antagonist capsazepine (CPZ, 10 μM) derived from the backbone of capsaicin [27]. Introduction of CPZ alone failed to alter either ST-EPSCs (Fig 4C and 4E) or sEPSCs (Fig 4C and 4D) indicating a lack of tonic endovanilloid activation of TRPV1. In the presence of CPZ, dynasore similarly increased sEPSC rates but CPZ effectively prevented the dynasore-induced blockade of evoked ST-EPSCs (compare Fig 1D to Fig 4C). The time course of facilitated sEPSC frequency by dynasore was generally similar to responses without CPZ (Fig 1). On average (n = 3), dynasore increased the rate of sEPSCs and washing failed to completely reverse that elevation (Fig 4D; dynasore: p = 0.003; Wash: p = 0.005; RM-ANOVA). In contrast to responses without CPZ, introduction of dynasore did not increase ST-EPSC failures (Fig 4E; n = 3; Dynasore vs. CPZ: p = 1; Wash vs. CPZ: p = 1; RM-ANOVA) and ST-EPSC amplitudes were unaltered (data not shown; n = 3; Dynasore vs. CPZ: p = 0.19; RM-ANOVA). The TRPV1 complex incorporates three distinct gating mechanisms (thermal, pH, and vanilloid) and TRPV1 antagonists differ considerably in their ability to block each of the three gating mechanisms [28]. For comparison to CPZ, we tested the TRPV1 antagonist JNJ 17203212 (JNJ, 10 μM), an effective antagonist of both vanilloid and pH activation of TRPV1 [29]. In contrast to CPZ, JNJ prevented all actions of dynasore (Fig 5). On average (n = 3), sEPSC rates (Fig 5E; JNJ + Dynasore: p = 0.99; JNJ + Wash: p = 0.55; RM-ANOVA) as well as ST-EPSC failure rates (Fig 5F; JNJ + Dynasore: p = 0.92; JNJ + Wash: 0.92; RM-ANOVA) were unchanged. Also, the ST-EPSC amplitude did not significantly change with dynasore (data not shown, p = 0.69, RM-ANOVA). Overall, these results suggest dynasore acts on TRPV1 sites sensitive to JNJ.

**Fig 6:**
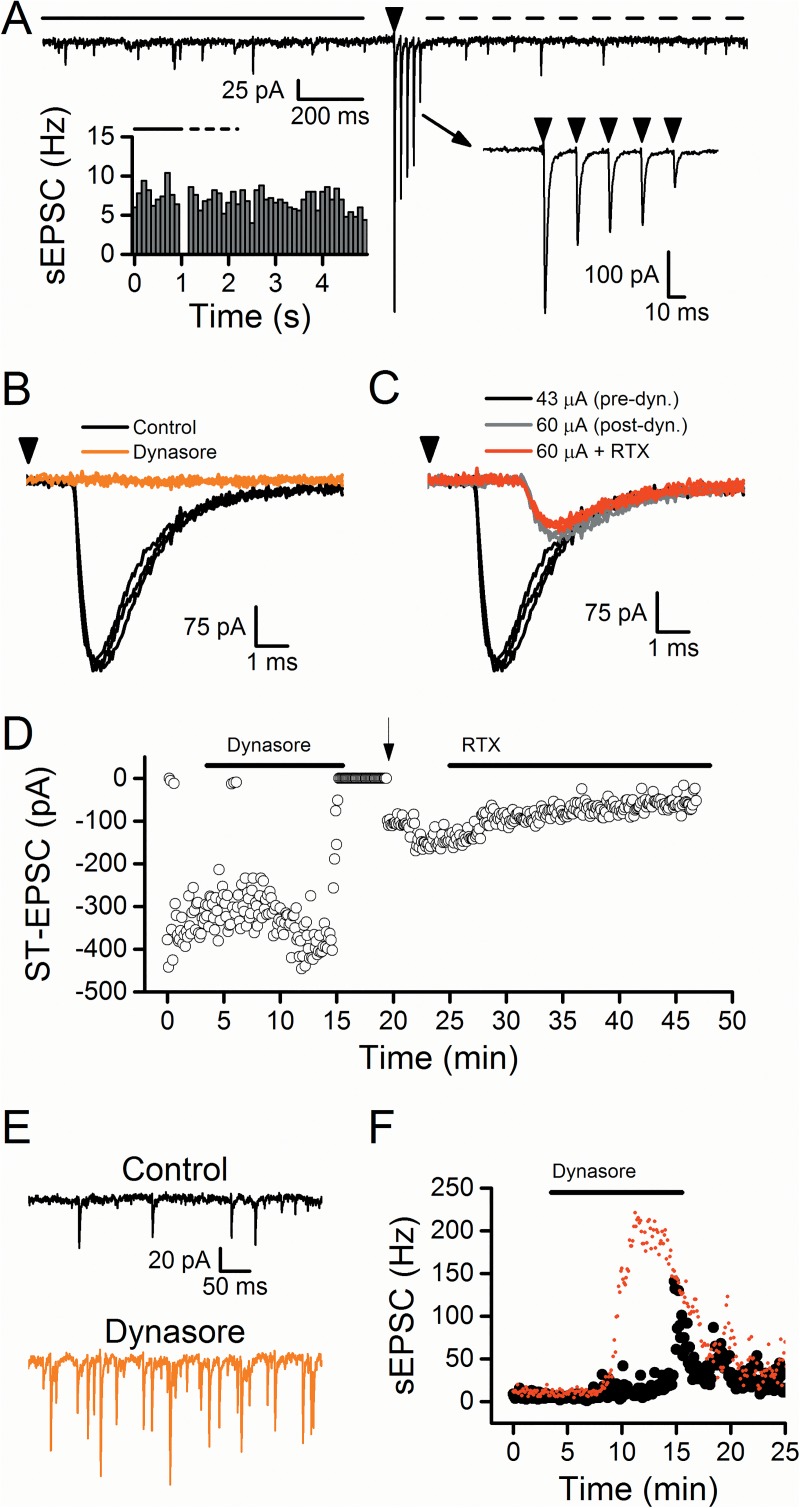
Dynasore facilitated the rate of sEPSCs and blocked the ST-EPSC at TRPV1- afferents. All panels in this figure are from a single representative cell. A) Bursts of five stimuli at 50 Hz (arrowhead) did not result in a transient increase in the spontaneous activity over the 1 s after stimulation (dashed line) compared to the 1 s before stimulation (solid line). The left inset portrays this lack of asynchronous release (compare to Fig 1A left inset) indicative of the absence of TRPV1. The right inset shows the frequency dependent depression of the evoked ST-EPSCs. In B-C, the arrowhead represents the stimulation of the ST. B) Dynasore inhibited the ST-EPSC (Control, black; dynasore, orange). C) Increasing the stimulation intensity during dynasore washout recruited a smaller input with a longer latency (60 μA, gray) compared to control (43 μA, black). Application of RTX did not affect this smaller ST-EPSC (60 μA + RTX, red) indicative of a TRPV1- afferent. D) Diary plot portrays the inhibition of the ST-EPSC with dynasore application. Unlike at TRPV1+ afferents, increasing the stimulation intensity (arrow) recovered an ST-EPSC. RTX did not inhibit the ST-EPSC. E) Dynasore increased the sEPSC activity (Control, black; dynasore, orange). F) Diary plot demonstrating the increased sEPSC frequency following dynasore application at this TRPV1- afferent (black circles) compared to the TRPV1+ afferent from Fig 1 (red circles).

The overwhelming majority of neurons within NTS are second order neurons receiving TRPV1-expressing ST afferents [9]. We screened for TRPV1+ ST-NTS neurons by using the presence of asynchronous release as a criterion. A small number of NTS second order neurons have monosynaptic ST connections that lack TRPV1 expression and asynchronous release. In the course of our studies, we encountered a single case of a neuron lacking asynchronous release (Fig 6A). Using an identical protocol to other neurons, dynasore rapidly blocked ST-EPSCs in this neuron (Fig 6B and 6D). Attempting to restore the ST-EPSC, increased ST shock intensity successfully recruited a new, longer latency synaptic input (arrow, Fig 6D). The rescued ST-EPSC at higher shock intensity had a much smaller peak amplitude as well as the longer latency suggesting that it was not the same input initiated by the pre-dyn, low intensity activation (Fig 6C). RTX (1 nM) failed to alter ST-EPSC amplitudes of this newly recruited ST input consistent with an ST input lacking TRPV1. Dynasore increased sEPSC rate in this TRPV1- NTS neuron (Fig 6E and 6F) but seemingly to a lesser degree and a pronounced delay compared to neurons with TRPV1+ ST inputs (red points, Fig 6F). Such a result is consistent with actions of dynasore that are independent of TRPV1 and not consistent with a vesicle depletion mechanism.

## Discussion

Many neurons rapidly recycle vesicle membrane during bouts of synaptic fusions and vesicle release in synaptic transmission [8, 30, 31]. Dynasore can disrupt action potential dependent vesicular release through the inhibition of dynamin to block endocytosis. Here, we investigated how dynasore affected spontaneous, evoked, and asynchronous release from TRPV1 expressing ST axons in the NTS. Dynasore facilitated both spontaneous and miniature frequency, suggesting a presynaptic site of action to increase the probability of release while interrupting evoked ST-EPSC transmission in the same neurons. With no graded changes in evoked amplitudes, we found no evidence of the activity-dependent vesicle depletion that was expected and conclude that vesicle recycling in ST afferents likely has no dynamin sensitive step. The relative timing of the effects of dynasore and its form–increases in spontaneous release prior to ST-EPSC failures–were reminiscent of TRPV1 activation in these ST afferents. Prior application of TRPV1 antagonists brought mixed and puzzling results. CPZ prevented the increase in ST-EPSC failures but not the increase in sEPSC frequency, while JNJ prevented both actions of dynasore suggesting a link to TRPV1 receptors. However, dynasore blocked the ST-EPSC and increased the spontaneous rate at a neuron whose ST afferent input lacked TRPV1 suggesting that dynasore acts independent of TRPV1. Overall and across 13 neurons, our results consistently point to presynaptic actions with decreases in afferent action potential conduction into the ST terminal as well as a separate action which facilitates spontaneous glutamate release. These actions of dynasore at ST afferents do not fit with the canonical view of disruption of endocytosis.

The NTS contains three distinct glutamate signals at TRPV1+ ST afferents: evoked, spontaneous, and asynchronous release [9], and these forms of transmission stand on a solid foundation of work [4, 14, 16, 17, 19, 22, 32–34]. Each type of release depends on increases in calcium but from different sources: spontaneous release requires calcium influx via TRPV1 while evoked and asynchronous release depends on calcium increases via VGCCs [4]. In addition, the increases in calcium remain tightly controlled in micro/nano-domains such that calcium from TRPV1 activation only affects spontaneous release while calcium from VACCs only affects evoked and asynchronous release [4]. Each of these vesicle pools presumably depends on membrane recycling to maintain synaptic release during times of activation and vesicle turnover. Endocytosis requires clathrin-coated vesicle formation and dynasore can disrupt the critical role of the GTPase dynamin in this process [25]. We observed in NTS neurons that dynasore increased spontaneous release similar to reports in hippocampal cultures [8, 35] and frog neuromuscular junction [6]. In some reports, dynasore failed to completely block evoked release in other systems suggesting diversity in vesicle recycling machinery [6, 30, 36–38]. We did not observe any activity-dependent rundown of evoked amplitudes during dynasore [8]. The ST-EPSC block resembles a conduction block with no evidence of an accumulating decrease in the probability of release as observed with other presynaptic inhibitors, e.g. CB1 [19, 39], GABA_B_ [40, 41] or mu opioid [42, 43]. We conclude from our NTS study that recycling in ST afferent terminals does not have a dynamin dependent step and this conclusion extends across multiple forms of glutamate release from ST afferents. The distinctions between spontaneous and evoked release mechanisms in NTS neurons reinforces the idea that spontaneous and evoked release arise from independent mechanisms governing separate pools of vesicles [4, 14, 17, 19].

Perhaps the most puzzling aspect of the present studies is the mechanistic implication that the actions of dynasore can be interrupted by two well characterized competitive antagonists to the TRPV1 receptor. Intense TRPV1 activation triggers robust increases in basal sEPSC rates of release that is followed by subtle increases in ST-EPSC arrival times which ultimately terminates in full failures to evoked release irrespective of stimulus shock intensity [15, 16]. The augmented spontaneous release from TRPV1 activation arises from calcium entry through the TRPV1 channel with no contribution from action potential conduction or VACCs [4]. Globally, dynasore-induced synaptic changes resembled TRPV1 activation. Both CPZ and JNJ were used at concentrations which effectively antagonize RTX activation of TRPV1 and its vanilloid gating mechanism. Thus, the failure of CPZ to block dynasore increases in sEPSCs suggests that endovanilloid mechanisms are unlikely to contribute. Surprisingly, CPZ prevented the failures of the evoked response and yet the sEPSC rates increased with dynasore. JNJ had the additional effect of blocking the dynasore facilitation of sEPSCs, suggesting a potential role for dynasore to activate the pH sensitive domain of TRPV1 due to the ability of JNJ to block this site unlike CPZ [29]. However, the occurrence of a similar response profile in an ST-NTS neuron lacking TRPV1, increased spontaneous release and blocked evoked release, undermines the idea that TRPV1 expression is even required. Additional surprises included the inability for RTX to activate TRPV1 following dynasore washout implying long lasting dynasore-induced action which disabled vanilloid activation of TRPV1. The interactions of RTX and TRPV1 antagonists with dynasore are without ready explanation.

Our report on NTS transmission joins other reports of dynamin-independent effects of dynasore [44]. The dynasore induced increase in spontaneous release at ST afferents likely relies on an increase in presynaptic calcium. Dynasore rapidly increased cytosolic calcium in rat pulmonary microvascular endothelial cells [45], and it is reported to deplete membrane cholesterol and disrupt lipid rafts [46]. Cholesterol and lipid raft disruption decreased TRP function [47, 48], effects opposite of those we observed, while depletion of membrane cholesterol increased spontaneous release and decreased evoked release in cerebellar [49] and hippocampal [50] cultured cells, results consistent with our observed effects of dynasore. Disruption of membrane cholesterol and perhaps lipid rafts might upset highly compartmentalized aspects of membrane excitability and microdomains important to differential signaling in ST synaptic endings [44].The diversity of results reported in the literature make it difficult to draw parallels confidently to our observations in NTS. Thus, our experiments suggest that dynasore does not inhibit endocytosis in the NTS but instead dynasore had clear, reproducible actions that appear to be dynamin-independent.
